# Impact of wine-grape continuous cropping on soil enzyme activity and the composition and function of the soil microbial community in arid areas

**DOI:** 10.3389/fmicb.2024.1348259

**Published:** 2024-02-13

**Authors:** Rui Song, Wen Zong Zhu, Hua Li, Hua Wang

**Affiliations:** ^1^College of Enology, Northwest A&F University, Yangling, Shanxi, China; ^2^China Wine Industry Technology Institute, Yinchuan, Ningxia, China

**Keywords:** continuous cropping, perennial crop, vineyard, metagenomic sequencing, soil enzyme activity

## Abstract

**Introduction:**

Continuous cropping affected the stability of soil enzyme activity and the structural characteristics of microbial community. Owing to challenges in the study of complex rhizosphere microbial communities, the composition and function of these microbial communities in farmland ecosystems remain elusive. Here, we studied the microbial communities of the rhizosphere of wine grapes with different years of continuous cropping and investigated their relationships with soil enzyme activity.

**Methods:**

Metagenomic sequencing was conducted on the rhizosphere soils from one uncultivated wasteland and four vineyards with varying durations of continuous cropping.

**Results:**

The predominant microbial were bacteria (98.39%), followed by archaea (1.15%) and eukaryotes (0.45%). Continuous cropping caused a significant increase in the relative abundance of *Rhizobiales* and *Micrococcales* but a marked decrease in *Solirubrobacterales*. At the genus level, 75, 88, 65, 132, and 128 microbial genera were unique to uncultivated wasteland, 5, 10, 15, and 20 years of continuous cropping, respectively. The relative abundance of genes with signal transduction function was the highest. The activity of all enzymes measured in this study peaked at 5 years of continuous cropping, and then decreased with 10 to 15 year of continuous cropping, but increased at 20 years again. In addition, soil enzyme activity, especially of alkaline phosphatase was significantly correlated with the diversity of the dominant microorganisms at the genus level. Moreover, the coupled enzyme activities had a greater impact on the diversity of the microbial community than that of individual enzymes.

**Conclusion:**

Our findings reveal the composition and function of the soil microbial communities and enzymes activity in response to changes in cropping years, which has important implications for overcoming continuous cropping obstacles and optimizing land use.

## 1 Introduction

The global impact of the vine and wine industry in terms of production, acreage, and trade is widely recognized. In recent years, with the adjustment of the planting structure and the rapid development of the agricultural economy in China, there has been a consistent increase in the area under cultivation for wine production, accompanied by an accelerated shift toward intensive large-scale intensive production. However, continuous cropping remains a common practice in vine cultivation due to geographical factors and the inherent characteristics of the crop. Overcoming barriers associated with continuous cropping has been a primary focus of research on perennial cash crops (Gao et al., 2019; Ledo et al., 2020; Pang et al., 2021). The obstacles include depletion of soil nutrients, alterations in the composition and abundance of soil microbial species, and modifications to the fundamental structure of the soil (Sun et al., 2021; Zhang et al., 2021). The aggravation of diseases, insect pests, tree weakness, replanting, and regeneration caused by continuous cropping reduced the quality and yield of crops (Chen et al., 2020; Betancur-Agudelo et al., 2021). The obstacles were intricately linked with an imbalance within the structure of the soil microbial community (Zhang Y. et al., 2019; Liu et al., 2021). Therefore, the relationship between microbial community structure and function in the rhizospheric soil and continuous cropping obstacles has attracted increasing attention.

Microorganisms are the most active component of soil ecosystem, which can make timely response and feedback to soil ecosystem (Toledo et al., 2023; Zhao et al., 2023). The diversity and composition of soil microbial communities play a crucial role in preserving soil health and quality. A higher abundance of bacteria and fungi in the soil was positively correlated with the overall condition of the soil ecosystem (Liu et al., 2015). However, continuous cropping practices have been found to exert numerous detrimental effects on soil microorganisms. For instance, continuous cropping practices led to a significant decline in functional strains such as aerobic and nitrogen-fixing bacteria, thereby disrupting the original structure of the microbial community of the soil and subsequently impacting plant growth (Wang et al., 2018). In addition, they can inhibit the secretion of antibiotics by bacteria and enhance the proliferation of pathogenic bacteria, thereby leading to an elevation in the incidence of plant diseases (Zhang et al., 2020). Simultaneously, harmful microorganisms secrete secondary metabolites that attract beneficial microorganisms for their own benefit, further disrupting the soil microecology and driving it toward a state conducive to their survival, thus exacerbating the challenges associated with cropping. Therefore, investigating the impact of wine grape continuous cropping on the soil microbial and biochemical characteristics is highly significant for enhancing vineyard cultivation practices and improving the soil microecological environment.

Soil enzyme activity (SEA) is considered a crucial indicator of the functioning of the soil ecosystem. It is generated through the biological activities of the soil fauna, organisms, and plant roots. Enzymes are proteins with catalytic properties that can significantly accelerate the biological reactions in organisms by a hundred- or even thousand-fold. Moreover, they play a vital role in the C, N, and P cycles within the soil (Fu et al., 2017; Sanchez-Hernandez et al., 2017). The SEA reflects the trend and intensity of various biochemical processes in the soil, making it more sensitive than changes to the physicochemical properties. Therefore, compared to the physical and chemical indices, ascertaining the changes in the SEA is a more intuitive and reliable approach to elucidate the impact of continuous cropping on soil quality (Li et al., 2023). In recent years, numerous analyses have been conducted analyses on the impact of continuous cropping on SEA; however, the findings were inconsistent. Some studies indicated a peak in SEA with increasing years of planting (Zhang et al., 2021), while others demonstrated a negative correlation between the two (Wang et al., 2022). These discrepancies may be attributed to variations in soil type and plant species. Furthermore, microbial communities play a significant role in maintaining the microecological environment of plant roots through their influence on SEA.

Although several studies have focused on the effects of continuous cropping of wine grapes on grape diseases and fruit quality (Merot et al., 2020; Wang B.T. et al., 2022), such research is scarce on its effects on the structure of the microbial communities of the rhizosphere and biochemical characteristics of the soil. To analyze the evolutionary trends in SEA and the microbial community associated with wine grapes under continuous cropping conditions, metagenomic sequencing was performed on the rhizosphere soil of one uncultivated wasteland and four vineyards under different durations of continuous cropping. This study aimed to achieve three primary objectives: (1) To investigate the impact of wasteland use and continuous cropping of wine grapes on SEA; (2) To elucidate the alterations in the diversity and structure of the microbial community associated with wine grapes under continuous cropping conditions; (3) To unravel the interaction between SEA and the microorganisms. In conclusion, this study aimed to provide a theoretical foundation for mitigating challenges associated with the continuous cropping of wine grapes while holding significant practical implications for guiding agricultural practices.

## 2 Materials and methods

### 2.1 Field experiments and soil sampling

**FIGURE 1:**
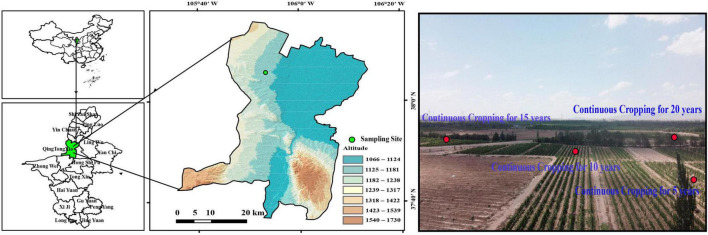
Distribution of study site.

The rhizosphere soil samples analyzed in this study were collected from the YaDai chateau of wine grape producing region at the eastern foot of the Helan Mountains in Ningxia (105°43′45′′–106°42′50′′ E, 37°28′ 08′′–37°37′23′′ N), China (Figure 1). The first batch of grapes was planted in 2,000. With the continuous expansion of the planting scale, the initial planting of 40 ha increased to 73.3 ha over time. This region features a continental arid climate characterized by low rainfall, dry conditions, abundant sunlight, and significant diurnal temperature fluctuations conducive to producing high-quality wine grapes. The average annual temperature was 9.2°C, with a daily difference ranging from 12 to 15°C. Precipitation levels ranged from 150 to 240 mm, while sunshine duration ranged from 1700 to 2,000 h⋅*a*^−1^. The predominant soil types included lime-lime, aeolian sand, and irrigated silt. Organic fertilizers were applied once a year between March and April, while other fertilizers such as N (38 kg⋅ha^–1^), P (30 kg⋅ha^–1^), and K (8 kg⋅^–1^) were applied between June and September. Additionally, the annual irrigation water volume was 3,900 m^3^⋅ha^–1^.

Soil samples were obtained from the topsoil (0–20 cm) in the rhizosphere of Cabernet Sauvignon grapes grown continuously for 5 (C5), 10 (C10), 15 (C15), and 20 years (C20). The 5-year-old vineyard was cultivated in April 2016, and 10-year-old vineyard, 15-year-old vineyard, and 20-year-old vineyard were cultivated in April 2011, April 2006, and April 2001, respectively. In addition, to determine the quality, microbial abundance, and composition of the microbial community of the soils used for cultivating grapes before reclamation, a sample was taken in the same manner as the control treatment from uncultivated lands (UL) within 500 m of the trial vineyards. The soil of the vineyard before reclamation was consistent with that of the wasteland soil. The wasteland has few crops grew and no agronomic management was done. Five 10 m × 10 m sampling sites were selected in each study plot at a distance of 100 m from each other. After removing the surface litter, soil samples were collected from the rhizosphere (0–20 cm) using a steel drill with a diameter of 5 cm. Five rock cores were randomly obtained at each sampling site and combined to form a composite sample. Fine roots were separated using a 2 mm sieve. All soil samples were transferred to sterile containers and transported to the laboratory in a refrigerator. Subsequently, a sterilized tube was used to store 10 g soil samples at −80°C for subsequent DNA extraction. Other soil samples were utilized for analyzing the SEA. Each experimental treatment was performed in five replicates.

### 2.2 Analysis of SEA

The fresh soil was dried under ventilation, and the SEA was analyzed using dry soil samples. SEA was determined following the method described by Guan (1986). Catalase activity was determined by potassium permanganate titration, with 0.3% hydrogen peroxide solution as the substrate, under acidic conditions, with 0.1N potassium permanganate titration, residual hydrogen peroxide was determined. Catalase activity was expressed as 0.1N potassium permanganate milliliters consumed by 1 g of soil after 20 min (ml⋅KMnO_4_⋅g^–1^⋅20 min^–1^); Alkaline phosphatase activity was determined using phenylene disodium phosphate colorimetry, based on the content of phenol released by soil sample and borate buffer (pH 10) cultured at 37°C for 24 h. Alkaline phosphatase activity was expressed as the amount of phenol released in 1 g of soil after 24 h (mg⋅d^–1^⋅g^–1^); Urease activity was determined by sodium phenolate-sodium hypochlorite colorimetric method, based on the content of ammonia released by soil samples cultured with citric acid buffer (pH 6.7) and 10% urea solution at 37°C for 24 h. Urease activity was expressed as NH_3_-N content in 1 g of soil after 24 h (mg⋅d^–1^⋅g^–1^); β-glucosidase activity was determined by nitrophenol colorimetry. The content of p-nitrobenzene-β-D-glucopyranoside solution was hydrolyzed to produce p-nitrophenol. β-glucosidase activity was expressed as the content of p-nitrophenol released in 1 g of soil every 1 h (ug⋅g^–1^⋅h^–1^); Amylase activity was determined by 3,5-dinitrosalicylic acid, based on the amount of maltose produced in 2% starch solution and acetate-phosphate buffer (pH 5.5) at 37°C for 24 h. Amylase activity was expressed as the content of maltose contained in 1 g soil after 24 h (mg⋅d^–1^⋅g^–1^); Sucrase activity was determined by 3,5-dinitrosalicylic acid based on glucose content in 8% sucrose solution and phosphate buffer (pH 5.5) at 37°C for 24 h. Sucrase activity was expressed as the amount of glucose contained in 1 g soil after 24 h (mg⋅d^–1^⋅g^–1^); Cellulase activity was determined by 3,5-dinitrosalicylic acid based on the amount of glucose produced after 72 h incubation of 8% sucrose solution and phosphate buffer (pH 5.5) at 37°C for 24 h. Cellulase activity was expressed as the amount of glucose contained in 1 g soil after 72 h (mg⋅d^–1^⋅g^–1^). The main instruments used include a ultraviolet spectrophotometer (Shimadzu, UV-1900i) and a full thermostatic oscillation box (China Instrument manufacturing Co., Ltd., GWQ-12).

### 2.3 Soil DNA extraction and metagenomic sequencing

Following the manufacturer’s recommendations, total microbial genomic DNA samples (The quality of samples was shown in Supplementary Figure 1) were extracted using the OMEGA Soil DNA Kit (Omega Bio-Tek, USA) and kept at −20°C for further analysis. A NanoDrop ND-1000 spectrophotometer (Thermo Fisher Scientific, Waltham, USA) and agarose gel electrophoresis were used, respectively, to evaluate the amount and quality of DNA. Metagenome shotgun sequencing libraries were constructed using the Illumina TruSeq Nano DNA LT Library Preparation Kit (Illumina, USA) with insert sizes of 400 bp. Sequencing data were obtained using an Illumina NovaSeq platform (Illumina, USA) with the PE150 strategy at Personal Biotechnology Co., Ltd. (Shanghai, China).

### 2.4 Metagenomic assembly and gene annotation

The raw sequencing data was processed to obtain high-quality reads for subsequent analysis using fastp (v0.23.2, -l 50 -g -W 5 -5 -q 20 -u 30) (Chen et al., 2018). The samples were assembled using Megahit (v1.2.9, default parameters) with the default parameters for meta-large datasets with contigs longer than 300 bp left (Li et al., 2015). The classification of the lowest common ancestor of contigs is performed by comparing them against the NCBI-nt database using mmseqs2 in “taxonomic” mode (Steinegger and Söding, 2017), while removing the configuration assigned to Viridiplantae or Metazoa for subsequent analysis. The prodigal (V2.6.3, -p meta) tool is utilized for predicting gene overlap groups. The CDS sequences of all samples were clustered using mmseqs2, employing the “easy-cluster” mode. A protein sequence recognition threshold of 0.90 was set, with a low utilization rate of covering residues at 90%. The abundance of these genes was assessed by mapping the readings from each sample onto the predicted gene sequence using featureCounts (v2.0.3), in order to determine the abundance of each gene (Corley et al., 2019). The functionality of the non-redundant genes were obtained by annotated using mmseqs2 against the protein databases of KEGG (Kanehisa and Goto, 2000), EggNOG (Huerta-Cepas et al., 2019), respectively.

### 2.5 Data analysis

The distribution of samples was mapped using Adobe Illustrator 27.4 (Adobe Inc., USA). SPSS Statistics R26 (IBM Corp., USA) was used for analysis of variance (ANOVA) to investigate the significance of the variability in soil enzyme activities across the various locations based on Waller-Duncan test and Tukey test. Non-metric multi-dimensional scale (NMDS) analysis based on Bray-Curtis distance and hierarchical clustering was performed to identify differences in microbial community composition in relation to wine grape continuous cropping, this analysis was performed by “Vegan” package in R 3.2.5. Heatmap showing the differences between the dominant microorganisms and the differences function genes of all sampling sites, this analysis was performed by the “pheatmap” package in R 3.2.5. Mantel test was used to analyze the correlation between dominant microbial diversity at genus level and soil enzyme activity, this analysis was performed by “ade 4 1.7.13” package in R 3.2.5. Correlation networks analyzed co-occurrence relationships between functional genes, this analysis was performed by Python. Additionally, variance partitioning analysis (VPA) was employed to identify the contribution of soil enzyme activity variables to the diversity of predominant microbial community, this analysis was performed by “Vegan” package in R 3.2.5. Statistical significance was indicated by *P* < 0.05.

## 3 Results

### 3.1 Effects of wine grape continuous cropping on SEA

The continuous cultivation of wine grapes had significantly impacted the enzyme activities in different soils. The activities of seven enzymes ascertained in this study exhibited a remarkable enhancement at C5, followed by a decline with increasing planting years (Table 1). However, at C20, the enzyme activity again showed an upward trend. Notably, the activities of the soil urease, sucrase, cellulase, and catalase were significantly lower at C10 and C15 than at UL, indicating that continuous cropping negatively impacted the SEAs over a certain period.

**TABLE 1:** Effects of wine grape continuous cropping on SEA.

Soil enzyme activity	UL	C5	C10	C15	C20
Urease(mg⋅d^–1^⋅g^–1^)	0.542 ± 0.005b	1.400 ± 0.204a	0.302 ± 0.079c	0.340 ± 0.053c	1.253 ± 0.015a
Alkaline phosphatase(mg⋅d^–1^⋅g^–1^)	0.521 ± 0.004b	1.801 ± 0.102a	0.647 ± 0.117b	0.532 ± 0.085b	1.706 ± 0.309a
Amylase(mg⋅d^–1^⋅g^–1^)	4.400 ± 0.034c	4.734 ± 0.200bc	5.005 ± 0.275b	4.754 ± 0.151bc	6.095 ± 0.499a
Sucrase(mg⋅d^–1^⋅g^–1^)	6.819 ± 0.050c	11.691 ± 1.755b	4.061 ± 0.434d	5.350 ± 0.321cd	15.465 ± 2.191a
Cellulase(mg⋅d^–1^⋅g^–1^)	6.284 ± 0.048c	11.241 ± 1.704b	3.503 ± 0.996c	3.861 ± 0.769c	14.311 ± 2.847a
β-glucosidase(ug⋅g^–1^⋅h^–1^)	43.336 ± 0.481b	65.930 ± 9.071a	44.717 ± 4.247b	44.585 ± 5.146b	69.504 ± 3.270a
Catalase(ml KMnO_4_⋅g^–1^⋅20 min^–1^)	1.766 ± 0.006c	2.654 ± 0.238a	0.890 ± 0.291d	0.928 ± 0.082d	2.184 ± 0.174b

C5, C10, C15, and C20 represent the soil samples from fields that have been continuously growing wine grapes for 5, 10, 15, and 20 years, respectively, while UL represents uncultivated soil samples. Different letters in the table were indicated significant differences (*p* < 0.05, *n* = 5).

### 3.2 Effects of continuous cropping on the abundance and composition of soil microorganisms

**FIGURE 2:**
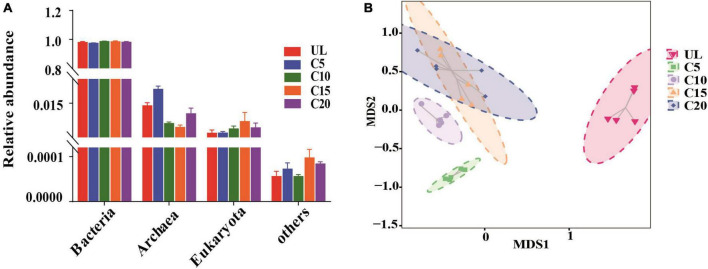
Effects of continuous cropping on the classification and composition of soil microorganisms were investigated. **(A)** The classification of bacteria, archaea, eukaryotes, and other organisms in different sampling sites was analyzed (*n* = 5). **(B)** Non-metric multi-dimensional scale (NMDS) analysis based on Bray-Curtis dissimilarities revealed significant species-level variations in soil microbial composition across different sampling sites. The stress value is an indicator reflecting the suitability of the model. It is generally believed that the stress value < 0.1 indicates that the model is reasonable.

In total, 99.99% of the microbial communities in the soils of the five sampling sites were identified, with bacterial communities dominating (average relative abundance of 98.39%), followed by archaea (1.15%), eukaryotes (0.45%), and others (unclassified sequence) (Figure 2A). The relative abundance of the bacterial communities was not significantly different among the five plots. The relative abundance of archaea was the highest at C5, followed by C10, C15, and C20, than that at UL. The relative abundance of eukaryotes was highest at C15, followed by C20 compared to UL. Furthermore, the microbial composition of the five plots displayed marked differences at the species level (Figure 2B).

**FIGURE 3:**
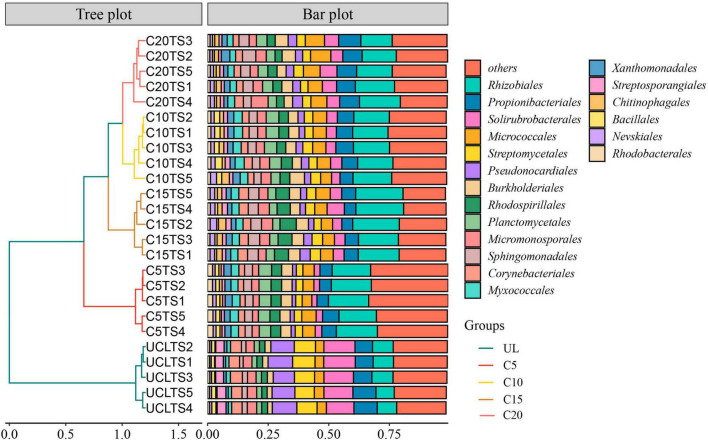
Hierarchical clustering analysis was performed on the relative abundance of soil microorganisms (Order level) based on Bray-Curtis distance.

Among the microorganisms at the five soil sampling sites, the main order of microorganisms were *Rhizobiales*, *Propionibacteriales*, *Solirubrobacterales*, *Micrococcales*, and *Streptomycetales* (Figure 3). A hierarchical clustering analysis of the relative abundances of soil microorganisms based on Bray-Curtis distance indicated a conspicuous variation in the microbial abundance between the wasteland and vineyard soils. Compared with UL, the relative abundances of *Rhizobiales* and *Micrococcales* were elevated after land use, whereas that of *Solirubrobacterales* and *Streptomycetales* decreased. In addition, after the reclamation of wasteland for the cultivation of wine grapes, the relative abundance of soil microorganisms in vineyards with varied planting years also showed marked differences.

**FIGURE 4:**
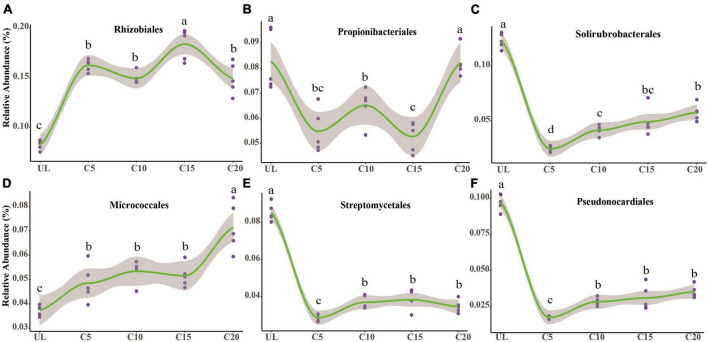
The impact of continuous cropping of wine grape changed the relative abundance of the dominant soil microorganisms at the order-level. The relative abundance of Rhizobiales **(A)**, Propionibacteriales **(B)**, Solirubrobacterales **(C)**, Micrococcales **(D)**, Streptomycetales **(E)**, and Pseudonocardiales **(F)** was altered due to long-term monoculture of wine grapes. A non-linear relationship was observed between the relative abundance of dominant microorganisms in the soil and the number of years of continuous cropping for wine grapes. The solid line represents the trend, while the gray area indicates a 95% confidence interval. Different letters indicate significant differences (*p* < 0.05, *n* = 5).

**FIGURE 5:**
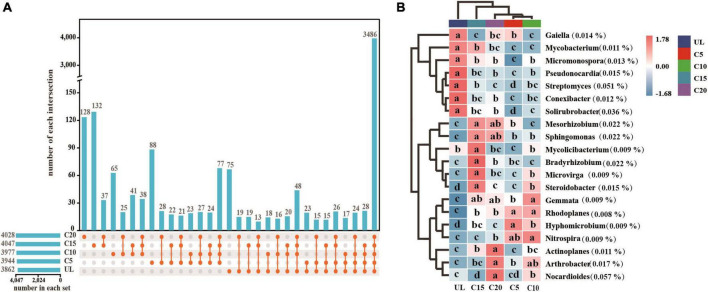
Effect of continuous cropping on the dominant genera of soil microorganisms. **(A)** Upset diagram showing the characteristics of common and endemic microorganisms at the genus level; **(B)** Heatmap showing the differences between the dominant microorganisms at the genus level. Lower letters indicate significant differences in the relative abundance of genus level dominant microorganisms (*P* < 0.05).

After the reclamation of wasteland for vineyards, the relative abundances of *Rhizobiales* enhanced appreciably (Figure 4A), indicating that the rhizospheric soil environment was conducive to their survival. The relative abundance of *Propionibacteriales* was the lowest at C15 (Figure 4B). However, the relative abundance of *Solirubrobacterales*, *Streptomycetales*, and *Pseudonocardiales* reduced significantly after reclamation but increased with the cropping years (Figures 4C, E, F). The relative abundance of *Micrococcales* was the lowest at UL but increased due to long-term continuous cropping (Figure 4D). Metagenomic analysis identified 3,862, 3,944, 3,977, 4,047, and 4,028 microbial genera in UL, C5, C10, C 15, and C20, respectively. Among them, 75 88, 65, 132, and 128 microbial genera were unique to UL, C5, C10, C15, and C20, respectively (Figure 5A). Heatmap analysis further clarified the differences in the abundance of the top 20 dominant genera microorganisms under continuous cropping. *Nocardioides*, *Streptomyces*, and *Solirubrobacter* had the highest relative abundance (Figure 5B). Specifically, the relative abundance of *Streptomyces* and *Solirubrobacter* had the highest relative abundance at UL and varied in abundance across years of continuous cropping, while *Nocardioide* was highest at C20.

### 3.3 Effects of continuous cropping on soil microbial function

**FIGURE 6:**
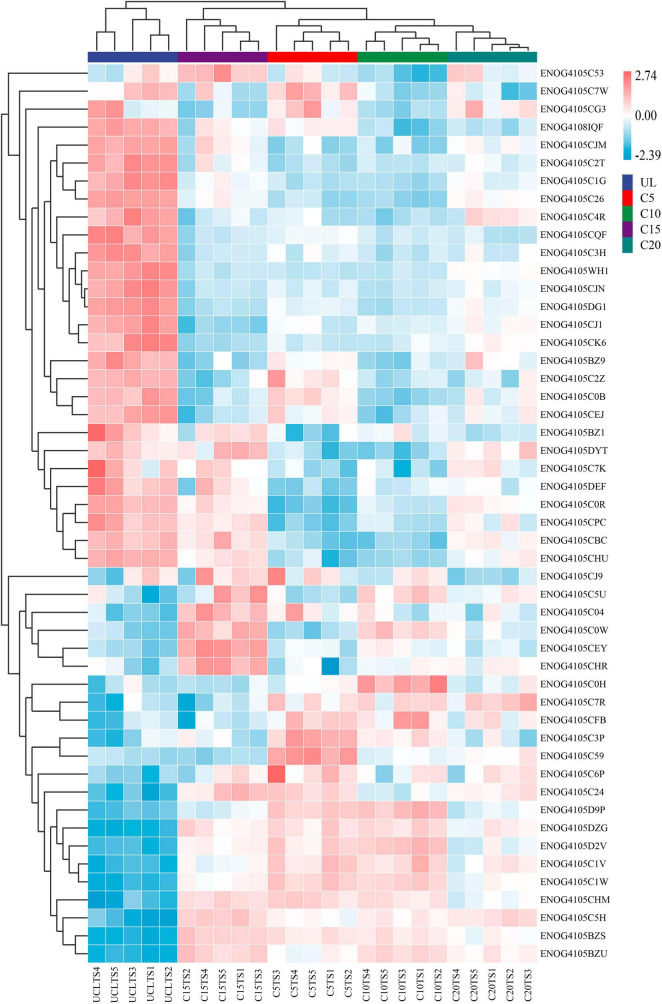
Heatmap of the abundance of functional genes (eggNOG, top50) across microbial communities of five soil sampling sites. The meaning of the functional gene database ID, description, class, and category shown in Supplementary Table 1.

Continuous cropping led to differences in the relative abundance of different functional genes in soil microorganisms. According to the sample cluster analysis of the heatmap, samples from the same continuous planting year were grouped together. UL was significantly different from other groups. Among them, the largest difference in the relative abundance of different functional genes between UL and C20 groups (Figure 6).

**FIGURE 7:**
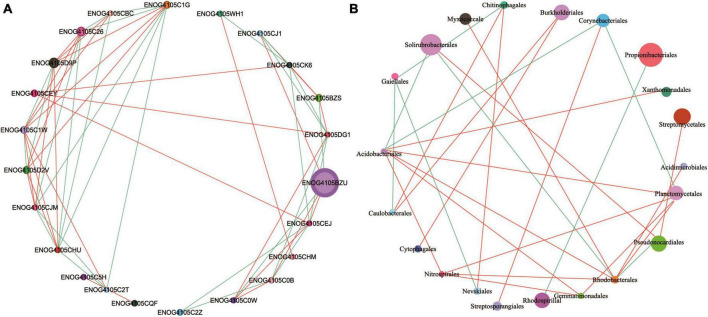
Correlation network diagram of Top 50 functional genes **(A)** and Top 30 genus level microbial **(B)** of five soil sampling sites. The lines color indicates whether the relationship is positive (red) or negative (green). The relationship are shown by connections between nodes (Spearman correlation coefficient, *r* = 0.6; *P* = 0.05). The abundance is directly proportional to the size of the node. The meaning of the functional gene database ID, description, class, and category shown in Supplementary Table 1.

The functional genes and community composition of soil microorganisms showed significant interaction. The abundance of ENOG4105BZU was much higher than that of other functional genes, and was positively correlated with ENOG4105CJ1, ENOG4105CEJ and ENOG4105C0B, and negatively correlated with ENOG4105BZS and ENOG4105C0W (Figure 7A). In addition, ENOG4105C1W, ENOG4105D2V and ENOG4105CHU all had complex network relationships and interacted with multiple functional genes. However, the network relationship of ENOG4105CQF is relatively simple and only positively correlated with ENOG4105C2T. Among the genera, *Propionibacteriales* with higher abundances were negatively correlated with *Rhodospirillal* (Figure 7B). *Solirubrobacterales* was positively correlated with *Pseudonocardiales* and negatively correlated with *Acidobacteriales* and *Rhodobacterales*.

### 3.4 Effects of SEA on microbial composition and functional genes

**FIGURE 8:**
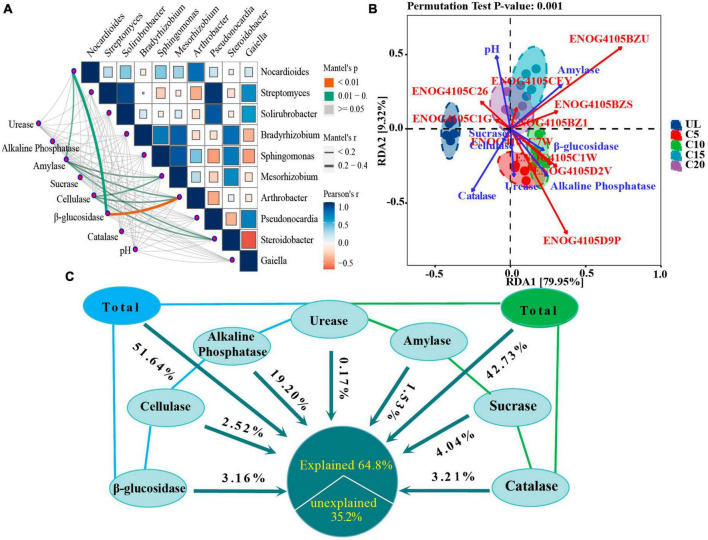
Effects of SEAs on microbial composition and functional genes. **(A)** Mantel test analysis showing the correlation between dominant microbial diversity at genus level and SEAs. **(B)** Redundancy analysis (RDA) revealed the effects of SEAs on functional genes. **(C)** VPA analysis showing the contribution of SEAs variables to the diversity of predominant microbial community. The meaning of the functional gene database ID, description, class, and category shown in Supplementary Table 1.

Continuous cropping led to alterations in the SEA and exhibited a significant association with the diversity of the dominant genera of microorganisms. β-glucosidase was extremely highly associated with the relative abundance of *Arthrobacter* (*p* < 0.01), and significantly associated with that of *Nocardioides* (*p* < 0.05). The activity of amylase and cellulase were significantly associated with the relative abundances of *Steroidobacter*, *Mesorhizobium*, and *Arthrobacter* (Figure 8A).

RDA analysis showed that SEAs played an important role in the abundance of functional genes in microbial communities. Explanatory variables (amylase, sucrase, β-glucosidase, catalase, alkaline phosphatase, cellulase, sucrase, and pH) accounted for 89.27% of the total variation in the model, with the first two axes accounting for 79.95 and 9.32%, respectively (Figure 8B). pH, catalase and amylase activity were the main environmental variables that affect the abundance of functional genes. In addition, the amylase activity was positively correlated with the abundance of genes that function as signal transduction mechanisms (ENOG4105BZU); pH was positively correlated with the abundance of genes with lipid transport and metabolic functions (ENOG4105CEY), and negatively correlated with the abundance of genes with energy production and conversion functions (ENOG4105C7W).

Variance partitioning analysis analysis determined the extent to which the variables of SEA contributed to the impact in the diversity of the dominant microbial communities. Alkaline phosphatase, sucrase, and catalase contributed the most to the effects on the variety of the dominant microbial communities at the genus level, which were 19.22, 4.04, and 3.21%, respectively (Figure 8C). However, the coupling relationship between the SEAs had a more significant impact on the microbial community diversity than that of the individual enzymes. The results showed that the combined effects of β-glucosidase, cellulase, alkaline phosphatase, and urease contributed 51.64%, and of urease, amylase, sucrase, and catalase 42.73% to these impacts on the diversity of the dominant microbial community.

## 4 Discussion

### 4.1 SEA was influenced by land use and varying durations of continuous cropping

The continuous cropping barriers are considered the phenomenon in which after the soil was continuously planted with the same or related crops for a long time, the growth of the crop was weakened even under normal conditions of management, resulting in reduced production and quality (Gao et al., 2019; Chen et al., 2021). Wine grapes, a crop with a high economic value and a long cultivation period with continuous cropping, are typical. Given that 7.3 million hectares of vineyards are planted in China, it is crucial to reveal the effects of long-term monoculture on the soil biochemical indices for improving the sustainable cultivation of wine grapes. SEA regulated the essential metabolic pathways of soil microorganisms and was frequently employed to characterize the soil quality and the diversity of the functional soil microbial communities (Zhang C. et al., 2019; Piotrowska-Długosz et al., 2022). Overall, the activities of the enzymes included in this study enhanced markedly after land use but were inversely proportional with the planting years, which was consistent with previous studies (Liu et al., 2021). The impact of continuous cropping on American ginseng was investigated, which remarkably reduced the Alkaline phosphatase and cellulase activities, which gradually returned to the pre-planting levels. This outcome may be attributed to the following three factors. Firstly, the sampling area selected for this study was in Ningxia, China, characterized by a typical continental climate, where vines were conventionally buried during winter to overcome the harsh conditions. This operation resulted in the depletion of soil organic matter and nutrients, leading to a decline in SEA. Secondly, the degradation products such as pesticides, disinfectants, and insecticides used in agronomic management inhibited the SEA (Niemi et al., 2009; Gao et al., 2020). Third, long-term monoculture may alter the diversity and composition of soil microbes, resulting in variations in microbial metabolic pathways and thus affecting enzyme activity (Piotrowska-Długosz et al., 2022). Urease is an enzyme that is closely associated with nitrogen metabolism and catalyses the breakdown of urea into ammonia and carbon dioxide (Zhang et al., 2013). The activity of urease was peaked at C5, then remarkably reduced at C10 and C15, compared to UL, which was in line with the growth pattern of wine grapes. When continuously planted for 10–15 years, wine grapes were in the vigorous growth stage, and the N consumption of plants was greater than the amount applied, which affected urease activity. Alkaline phosphatases are the most frequently studied soil enzymes because they are the fastest to react to environmental stress caused by anthropogenic and natural factors (Lemanowicz et al., 2020). The activities of this enzyme also exhibited an upward trend after C20, which could be attributed to the continuous cropping years and soil texture. The prolonged monoculture led to the stabilization of clay minerals (Chen and Arai, 2023), thereby reducing the enzyme activity rates in this region. Furthermore, soil microbes persistently secrete enzymes to promptly respond to the changes in substrate availability even under nutrient-deficient conditions (Chacon et al., 2006). Sucrase can promote the hydrolysis of carbohydrates, increase soluble nutrients in soil, and provide nutrients for the growth and reproduction of microorganisms. Its activity level can reflect the law of soil organic carbon transformation, decomposition and accumulation, and can reflect the intensity of soil biological activity (Lemanowicz et al., 2020). Compared with wasteland, the activity of sucrase was lower in 10 and 15 years of continuous cropping, it may be due to the loss of soil organic carbon caused by long-term monoculture, which inhibited the secretion and release of enzymatic substances by soil microorganisms, thus reducing soil enzyme activity. It may also be related to the change of soil physical and chemical properties such as soil pH (Kompała-Ba̧ba et al., 2020).

### 4.2 Continuous cropping of wine grapes changed the diversity and composition of the soil microbial community

The effects of continuous cropping of wine grapes on the rhizospheric microorganisms primarily encompassed growth inhibition and promotion. Among them, the inhibitory effect was predominantly observed as an obstacle to cropping, resulting from the elevated relative abundance of soil pathogens (Xin et al., 2022). The promotive effect was primarily demonstrated by enhancing the rhizospheric growth environment through modulation of the structure and diversity of the functional bacterial communities with growth-promoting potential (Liu et al., 2020; Wei et al., 2023). Continuous cropping had no significant effect on the relative abundance of bacteria, but there was a significant difference in the effects on archaea and eukaryotes (Figure 1A). This suggests that archaea and eukaryotes are more inclined than bacteria to adapt to alterations in the soil environment, primarily due to the widespread distribution of archaea and their frequent association with habitats characterized by unconventional physicochemical properties (Walsh et al., 2005). Archaea is more important than bacteria in driving soil stoichiometry in phosphorus deficient habitats (Wang J. T. et al., 2022). The main order of microorganisms were *Rhizobiales*, *Propionibacteriales*, *Solirubrobacterales*, *Micrococcales*, and *Streptomycetales*, The relative abundance of *Rhizobiales* significantly increased after land use, and the relative abundance varied across different planting years. *Rhizobiales* (Proteobacteria) represent a diverse and abundant microbiome taxa that establishes both mutualistic and pathogenic relationships with plants while playing a crucial role in nitrogen fixation processes (Clúa et al., 2018; Lemos et al., 2021). After the land use for vineyards, the litter and animal residues increase the nitrogen fixation of the soil, consequently leading to a higher relative abundance of *Rhizobiales*. In addition, the antagonism between *Rhizobiales* and pathogenic microorganisms also promoted the increase of their relative abundance (Ji et al., 2022). The relative abundances of *Solirubrobacterales* and *Pseudonocardiales* were the highest in the uncultivated soil and the lowest in the early stage of land use (C5). With the increase of cropping years, the relative abundances of *Solirubrobacterales* and *Pseudonocardiales* showed an increasing trend. This could be attributed to *Solirubrobacterales* and *Pseudonocardiales* have extremely diverse trophic modes, which change with different soil conditions, resulting in different relative abundances (Shange et al., 2012; Gummerlich et al., 2020).

Continuous cropping of wine grapes resulted in significant differences in the dominant genera of microorganisms. *Nocardioides*, *Streptomyces*, and *Solirubrobacter* revealed the highest relative abundance. The diversity of bacterial communities in the rhizosphere and block soils of Artemisia annua growing in the Ugandan highlands was dominated by the genera *Nocardioides* and *Solirubrobacter* (Aidah et al., 2023), which was similar to the findings of this study. *Nocardioides* is renowned for its ability to tolerate high salt concentrations and release phosphatases (Dastager et al., 2008). The relative abundance of *Nocardioides* varied, corresponding to the continuous cropping years, suggesting differential capabilities of degrading toxic substances in the soil. *Streptomyces* is a significant antagonist of soil pathogens (Dundore-Arias et al., 2019), exhibiting the highest relative abundance at UL. However, its relative abundance was reduced following land use, which could be potentially attributed to alterations in the soil environment during the transformation of wastelands to wine grape cultivation. Consequently, this change weakened its inhibitory capacity against pathogens.

### 4.3 Continuous cropping of wine grapes changed the function of soil microbial community

During the growth of wine grapes, vines residues and root exudates will be different, resulting in differences in soil microbial communities and functions. The relative abundance of different functional genes in UL and C20 was the largest, indicating that land use and wine-grape continuous cropping had changed the function of soil microorganisms. The ENOG4105BZU gene has the signal transduction function and exhibits the highest relative abundance, which is because the formation and transformation of soil organic matter are inseparable from the mutual transmission of signals and transduction information among soil microorganisms (Song et al., 2023). In addition, the microbial community is susceptible to soil disturbance and changes in the external environment, and needs timely feedback to respond to these changes, so the abundance of genes with signal transduction functions was highest. Under continuous cropping conditions, microbiomes have developed diverse stress response systems to maintain their functionality (Bei et al., 2023). In this study, most of the genes involved in inorganic ion transport and metabolism (ENOG4105CJM, ENOG4105C2T, ENOG4105C0R) were highly expressed in UL (Figure 6). However, after wine-grapes were planted, their expression decreased significantly. This suggests that the utilization of inorganic carbon sources may be a means for microbial survival in uncultivated and poor soils. After land use, chemotaxis enables microorganisms to move toward nutrients and sense environmental changes (Sourjik and Wingreen, 2012), which led to the expression of genes with signal transduction function (ENOG4105BZU, ENOG4105D9P, ENOG4105C1W) and carbohydrate transport and metabolism function (ENOG4105DZG) were increased.

Soil pH, amylase and catalase had the most significant effects on the relative abundance of functional genes (Figure 8B). Amylase was positively correlated with ENOG4105BZU (Signal transduction mechanisms), ENOG4105BZS (Defense mechanisms) and ENOG4105BZ1 (Defense mechanisms). This may be related to continuous cropping reduces soil enzyme activity and changes the pattern of microbial utilization of soil substrates, thus inhibiting the expression of related genes (Zhao et al., 2021). In addition, pH was negatively correlated with ENOG4105C1W (Signal transduction mechanisms) and ENOG4105D9P (Signal transduction mechanisms). It was further confirmed that genes with signal transduction function were adapted to enrich under acidic pH conditions, which was consistent with previous studies (Wang et al., 2023). This may be due to the strong microbial activity in acidic soil (Boer and Kowalchuk, 2001), which can accelerate the decomposition of organic matter and promote the expression of genes with signal transduction function.

The network relationships between soil microbial communities can promote or inhibit the expression of functional genes. ENOG4105C2Z has the functions of post-translational modification and protein turnover, and the interaction with other functional genes was relatively simple, such as it was negatively correlated with the gene with replication, recombination and repair functions (ENOG4105C0B), which may be related to soil pH. Soil pH has been recognized as the essential driver of microbial structure and function (Luan et al., 2023; Piton et al., 2023). One study have shown that functional gene adaptations related to energy metabolism and protein turnover are enriched in neutral pH conditions, and gene adaptations with signal transduction, complex compound degradation, and recombination and repair functions are enriched in acidic pH conditions (Wang et al., 2023), this explains why the two functional genes were inversely related. In addition, the correlation network diagram showed negative correlations among many functional genes, indicated that the variation trend of rhizosphere microbial function were different, since the function of microbial communities is regulated by plant adaptation (Angulo et al., 2022). This would help maintain a balance between beneficial and harmful microorganisms in monocultural soils (Wu et al., 2018).

Continuous cropping was changed soil enzyme activity, led to significant functional differences in microbial communities, indicated that the expression of functional genes was mainly regulated by plant adaptability. Among them, the function of rhizosphere microbial community was mainly affected by soil pH, which mean that the change of soil quality caused by continuous cropping will have an increasing impact on the gene function of soil microorganisms. Reveal the effects of continuous cropping on functional genes will contribute to our understanding of crop-soil-microbial interactions. Thus, more evidence is needed on the effects of continuous cropping on microbial functional genes, which should be the focus of future research.

### 4.4 Correlation between SEA and the diversity and composition of soil microbial community

The effects of continuous cropping of wine grapes regarding their associated microorganisms were primarily achieved through the assimilation and transformation of nutrients and the secretion of metabolites during plant growth (Zhang C. et al., 2019; Chen et al., 2021). The alteration in the resident soil microflora has been widely associated with variations in the physicochemical properties of soil, SEA, and crop type (Zhang et al., 2016; Liu et al., 2021; Fan et al., 2022). A synergistic interplay between abiotic and biotic factors frequently influences the diversity and composition of microbial communities. Continuous cropping caused changes in SEA, significantly correlated with the variety and composition of the dominant microbial genera in the soil. β-glucosidase is sensitive to soil management practices (Knight and Dick, 2004). In this study, β-glucosidase was highly associated with the relative abundance of *Arthrobacter* (*p* < 0.01) and of *Nocardioides* (*p* < 0.05) (Figure 5A). Amylase and cellulase are essential enzymes in the soil (Houfani et al., 2018), and are responsible for the decomposition of crop litter (Wu et al., 2023). The results of this study indicated that Amylase and cellulase were markedly correlated with the relative abundance of *Steroidobacter*, *Mesorhizobium*, and *Arthrobacter*. Alkaline phosphatase, sucrase, and catalase contributed the most to the correlation of the diversity of dominant microbial genera at 19.20, 4.04, and 3.21%, respectively. Moreover, the coupled SEAs had a higher impact on the diversity of the microbial community than the individual enzyme activities. In fact, the microbe-based assimilation of organic matter requires the synergistic action of multiple enzymes. For example, the effective conversion of lignocellulose from litter in farmland to monomer sugars requires the synergistic action of oxidase, peroxidase, and xylanase, which is why the coupling effect of multiple enzymes are greater than that of a single enzyme (Jiménez et al., 2014).

## 5 Conclusion

Continuous cropping as an inevitable agronomic practice in farmland management, which has a negative impact on soil enzyme activity. Additionally, continuous cropping led to changes in the structure and function of soil microbial communities. Archaea and eukaryotes were more easily adaptable to the soil environmental changes than bacteria, and the structure and function of microbial communities were mainly regulated by plant adaptability. Microbes were utilized diverse stress response systems to maintain their diversity, especially under continuous cropping conditions, led to an increase in the relative abundance of genes with signal transduction functions. Furthermore, Alkaline phosphatase, β-glucosidase and cellulase were the main environmental variables influencing the composition of microbial communities, while pH, Catalase and Amylase were the main environmental variables influencing the abundance of functional genes. These results illustrated that continuous cropping altered both microbial structure and function as well as differences in soil enzyme activity. Overall, this study provides a theoretical basis for alleviating obstacles with continuous cropping in farmland. Considering limited arable land area in arid regions, the effects of continuous cropping on soil quality need to be further deepened and expanded. We suggest that future studies should focus more on minority closely related microbial taxa in the rhizosphere, especially fungi, which play crucial roles in soil quality and plant adaptive regulation.

## Data availability statement

The original contributions presented in this study are included in this article/Supplementary material, further inquiries can be directed to the corresponding authors.

## Author contributions

RS: Conceptualization, Data curation, Formal analysis, Investigation, Methodology, Software, Writing—original draft. WZ: Formal analysis, Writing—review and editing. HL: Funding acquisition, Methodology, Project administration, Supervision, Validation, Writing—review and editing. HW: Project administration, Software, Supervision, Writing—review and editing.
