# Supplementary material for: Impact of wine-grape continuous cropping on soil enzyme activity and the composition and function of the soil microbial community in arid areas
Source: Front Microbiol. 2024 Feb 13;15:1348259. doi: 10.3389/fmicb.2024.1348259 (PMC10896694; doi:10.3389/fmicb.2024.1348259)
Supplement: Supplementary file 1 [file Image_1.pdf]

The rarefaction curve of each samples had already approached a saturation plateau (Fig. S1), which indicated that the sequencing library had reached saturation, and the results can truly reflect the sample condition.

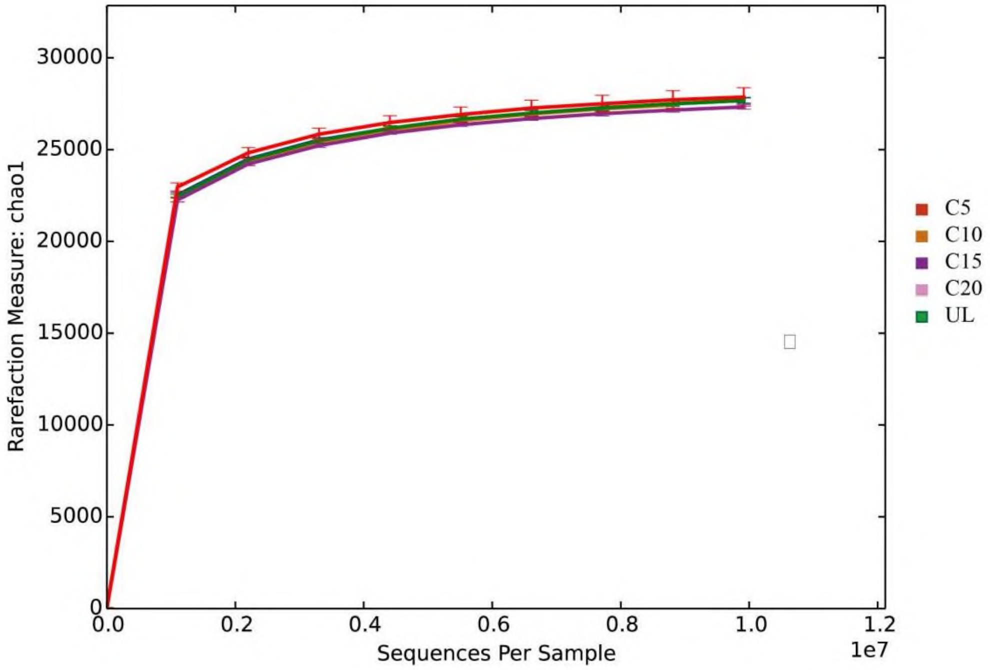

Figure S1. Rarefaction curves for all soil samples.
